# Increased microRNA-34c abundance in Alzheimer's disease circulating blood plasma

**DOI:** 10.3389/fnmol.2014.00002

**Published:** 2014-02-04

**Authors:** Shephali Bhatnagar, Howard Chertkow, Hyman M. Schipper, Zongfei Yuan, Vikranth Shetty, Samantha Jenkins, Timothy Jones, Eugenia Wang

**Affiliations:** ^1^Advanced Genomic Technology, LLCLouisville, KY, USA; ^2^Department of Neurology and Neurosurgery, McGill UniversityMontreal, QC, Canada; ^3^Memory Clinic, Department of Medicine, Jewish General HospitalMontreal, QC, Canada; ^4^Bloomfield Centre for Research in Aging, Lady Davis InstituteMontreal, QC, Canada; ^5^Division of Geriatric Medicine and Department of Clinical Neurosciences, Jewish General HospitalMontreal, QC, Canada

**Keywords:** peripheral blood mononuclear cells (PBMC), plasma microRNA, miR-34a, miR-34c

## Abstract

Circulating microRNAs, present either in the cellular component, peripheral blood mononuclear cells (PBMC), or in cell-free plasma, have emerged as biomarkers for age-dependent systemic, disease-associated changes in many organs. Previously, we have shown that microRNA (miR)-34a is increased in circulating PBMC of Alzheimer's disease (AD) patients. In the present study, we show that this microRNA's sister, miR-34c, exhibits even greater increase in both cellular and plasma components of AD circulating blood samples, compared to normal age-matched controls. Statistical analysis shows the accuracy of levels of miR-34c assayed by receiver operating characteristic (ROC) analysis: the area under the curve is 0.99 (*p* < 0.0001) and the 95% confidence level extends from 0.97 to 1. Pearson correlation between miR-34c levels and mild and moderate AD, as defined by the mini-mental state examination (MMSE), shows an *r*-value of −0.7, suggesting a relatively strong inverse relationship between the two parameters. These data show that plasma levels of microRNA 34c are much more prominent in AD than those of its sister, miR-34a, or than its own level in PBMC. Transfection studies show that miR-34c, as does its sister miR-34a, represses the expression of several selected genes involved in cell survival and oxidative defense pathways, such as Bcl2, SIRT1, and others, in cultured cells. Taken together, our results indicate that increased levels of miR-34c in both PBMC and plasma may reflect changes in circulating blood samples in AD patients, compared to age-matched normal controls.

## Introduction

Recently, microRNAs found in circulating blood, especially in cell-free plasma, have been noted functionally for inter-cellular and/or inter-organ communication. Circulating plasma microRNAs seem to be largely released from the cellular compartment, i.e., peripheral blood mononuclear cells (PBMC), either associated with specific protein or lipid molecules, or released in vesicles known as exosomes, with some portion derived from the cell debris of apoptotic bodies (Smalheiser, 2007; De Smaelea et al., 2010; Etheridge et al., 2011). Not surprisingly, differentially expressed plasma microRNAs have been noted as powerful biomarkers for several central nervous system disorders, from bipolar disorder to schizophrenia, Huntington's disease and Alzheimer's disease (AD) (Gaughwin et al., 2011; Rong et al., 2011; Suarez-Gomez et al., 2011; Geekiyanage et al., 2012; Sheinerman et al., 2012; Shi et al., 2012). Our own work has shown that lead microRNAs are differentially regulated in PBMC of AD patients compared with age-matched controls (Schipper et al., 2007; Maes et al., 2010). Therefore, unique microRNAs offer another aspect, in addition to specific transcriptome and serum protein profiling: blood biomarker discovery, for instance for AD.

Among many microRNAs, the miR-34 family, composed of three members, miR-34a, -34b, and -34c, is relatively well-understood. MicroRNA-34b, a plasma biomarker for Huntington disease (Gaughwin et al., 2011), and its sister, miR-34c, are linked as a bi-cistronic transcriptional unit (Liang et al., 2009); together with the other sister, miR-34a, they participate functionally in at least two signaling pathways: (1) Bcl2 for cell survival/apoptosis; and (2) SIRT1 deacetylase for p53 or neuroprotection signaling. SIRT1, p53, and miR-34a are involved in a positive feedback loop for miR-34a expression: acetylated p53 binds the promoter to activate this microRNA's transcription (He et al., 2007; Yamakuchi et al., 2008). Increased miR-34a suppresses SIRT1 expression, thereby diminishing its deacetylation of p53, leading to an increase in acetylated p53 transcriptional activity, resulting in the continued up-regulation of this microRNA (Yamakuchi et al., 2008).

Many reports, including our own work in aged mice, observe parallel changes during aging between differentially regulated circulating microRNAs and changes in the central nervous system (Li et al., 2011a). During aging, accumulating oxidative stress-activated p53 may tilt the balance toward age-dependent increase of miR-34a, observed in the brains of old rats and mice (Li et al., 2011b,c); and a reduction in age-dependent increase in miR-34a in brain has been observed in calorie-restricted mice (Khanna et al., 2011). In animal models of AD, increased miR-34a levels are observed in brains of mouse models bearing both the APP^swe^ and presenilin transgenes (Wang et al., 2009). MicroRNA 34-c, a sister of miR-34a, is also observed in the hippocampal region of AD animal models to be functionally connected to cognitive decline, because inhibition of this microRNA rescues memory impairment in AD transgenic mice, with corresponding regained SIRT1 levels (Zovoilis et al., 2011). These results suggest that increased expression of miR-34a and -34c may repress SIRT1 and Bcl2 expression, one of the many underlying causes for dysregulation of oxidative defense and neuronal cell survival in the brain of AD transgenic animals.

The main objective of this study is to test the hypothesis that unique microRNA changes in AD patients, specifically those of the microRNA-34 family, can be identified in circulating blood samples in both the cellular component, PBMC, and cell-free plasma. To test this hypothesis, we have expanded beyond our previous observation of increased miR-34a in PBMC of AD patients (Schipper et al., 2007) to include also miR-34c in our study of specimens of both blood components. Here, we report that miR-34c in circulating plasma and PBMC indeed exhibits increased levels of expression in AD patients, compared with age-matched normal elderly controls (NEC). Transfection study shows that miR-34c, similar to reported findings for miR-34a, functionally represses Bcl2, SIRT1, and other proteins, all key genes whose decreases are functionally associated with weakened oxidative defense and cell survival.

## Materials and methods

### Subjects and clinical evaluation

Informed consent was obtained from all participants, following the Institutional Review Board protocol approved by the Sir Mortimer B. Davis Jewish General Hospital (JGH) Research Ethics Committee. Blood samples were obtained from subjects at the Memory Clinic at the JGH, from 110 AD patients (age: 56–90) and 123 NEC (age: 61–90) without cognitive impairment. NEC were recruited by newspaper advertisements and public lectures, scored less than 4 on the Subjective Memory Scale of Schmand (Schmand et al., 1996), lacked other medical or neurological illnesses, and scored within 1 standard deviation (SD) of age and education means on memory screening tests. These consist of scores of 26 or more on the Montreal Cognitive Assessment (MoCA) (Nasreddine et al., 2005), a Mini-Mental State Examination (MMSE) scoring 25 or above (Folstein et al., 1975), and normal range scores on the delayed paragraph recall component of the Logical Memory Test of the WAIS-R (Wechsler, 2008). Patients with sporadic late onset AD were screened and assessed at the JGH McGill University Memory Clinic in Montréal; the diagnosis was made according to standard NINCDS-ADRDS criteria (McKhann et al., 1984), and conformed to the more recent revised criteria for probable AD as well (McKhann et al., 2011). Severity of dementia was stratified according to MMSE results: mild (21–24), moderate (10–20), or severe (4–9).

### Blood collection and isolation and quality evaluation of RNA samples

Approximately 30 ml of blood per donor was collected in EDTA vacutainers, and processed to isolate the plasma and PBMC fractions, using Ficoll-Plaque Plus (GE Healthcare, Piscataway, NJ). PBMC and plasma samples, stored in RNAlater buffer solution (Ambion, Austin, TX), were processed for RNA and protein isolation by Trizol/chloroform and centrifugation, to separate the upper RNA-containing aqueous phase from the lower proteinaceous phase, for isolation of microRNA from the plasma specimens, as previously described (Li et al., 2011a). Prior to RNA isolation, 0.625 ng of synthetic miRNA-39 from *Caenorhabditis elegans* (cel-miRNA-39) was added to the Trizol as a spike-in control for purification efficiency and cDNA synthesis quality validation during qPCR assays, as described below (Kroh et al., 2010) (Exiqon, #203203). RNA integrity was assessed using a Nanodrop spectrophotometer (Thermo Fischer Scientific, Barrington, IL) and Agilent 2100 bioanalyzer (Agilent Technologies, Waldbronn, Germany).

### Taqman MicroRNA real time qPCR

Plasma microRNAs were used to generate cDNA using the Taqman^®^ MicroRNA Reverse Transcription Kit (Applied Biosystems, Carlsbad, CA), with specific miRNA stem-loop primers for miR-34a, -34b, -34c, and -16, by MultiScribe Reverse Transcriptase; the reactions were carried out in a GeneAmp PCR System 9700 (Applied Biosystems). These cDNA samples were processed to assess mature miRNA levels by real time polymerase chain reactions (qPCR) using the Taqman^®^ Universal PCR Master Mix kit (Applied Biosystems). The qPCR was conducted in a 7500 real time PCR system (Applied Biosystems) under the following conditions: 95°C 10 min, 60 cycles of 95°C 15 s, and 60°C 1 min. The levels of mature cel-miR-39 mRNA were measured using individual TaqMan microRNA Assays (Applied Biosystems) according to the manufacturer's instructions (Exiqon, #203203); cel-miR-39 was used to normalize miRNA levels. A mean Ct was calculated for *C. elegans* miRNA for each sample, followed by calculating the median of all mean *C. elegans* synthetic miRNA Cts, taking all samples into consideration. Then a normalization factor was calculated for each sample by subtracting the mean *C. elegans* synthetic miRNA Ct of the sample of interest from the median value calculated earlier. This normalization factor was then integrated into the calculation of the raw Ct value obtained for each sample, which was further normalized by reference to Ct values for miR-16.

### Western blot analyses

Western blot analyses were performed as previously described (Bates et al., 2010; Li et al., 2011c), using an actin band for transfection cell lysates, to check equal loading across all lanes (Pendyala et al., 2010). The antibodies used were mouse anti-Bcl2 (1:1000, 692, Abcam Inc., Cambridge, MA), rabbit anti-Psen1 (1:500, 71181;Abcam), rabbit anti-Onecut2 (1:500, 28466;Abcam), rabbit anti-SIRT1 (1:500, 110304;Abcam), and rabbit anti-β-actin (1:1000, 8226;Abcam). Goat anti-mouse (31403, Thermo Fischer Scientific, Barrington, IL) was used for Bcl2 (1:2000), and goat anti-rabbit (31460, Thermo Scientific) for β-actin, SIRT1, Psen1, and Onecut2 (1:1000) as secondary antibodies. Intensities of antibody reactive bands were detected by Enhanced Chemiluminescence (ECL), (Pierce Biotechnology, Rockford, IL), and quantified by densitometry using ImageJ software (Public domain, NIH, USA).

### Construction of hsa34a and hsa34c GFP recombinants, and functional target suppression study

Micro-34a and -34c were amplified from DNA purified from human embryonic kidney cells (HEK 293 cell line; ATCC# CRL 1573) with the following primers:

miR-34a forward 5′-tctagaGAG TCC CCT CCG GAT GCC GTG,
reverse 5′-ggatccCCA CCC ACCG TGG CGC AG, 229 bp;
miR-34c forward 5′-tctagaAGC CCC TCC ATC CAT GTA ACG GT,
reverse 5′-ggatccAAC ACC CCT CTT CCC CAC GCA, 328 bp.

Amplified PCR products were purified and cloned by the Qiagen PCR Cloning kit (Qiagen, Valencia, CA), and subcloned into the pCDH-CMV-MCS-EF1-copGFP vector (System Biosciences, Mountain View, CA), which was then used for functional assays by transfecting human embryonic kidney cells (HEK 293), as previously described (Bates et al., 2010).

### Statistical analyses

All statistical analyses were performed using MS Excel 2010, SPSS 17.0 statistical software package (IBM), or SAS version 9.2 (SAS Institute Inc., Cary, NC). Student's *t*-test and one-way analysis of variance (ANOVA) were performed to determine significant differences for two-way or more than two groups. For multiple comparisons, Fisher's Least Significant Difference (LSD) test was used, following one-way ANOVA, in order to assign statistical significance, and then the Scheffé test was applied to reduce Type 1 errors. Levels of miR-34c, miR-34b, and miR-34a were normalized with regard to miR-16, as recommended by the manufacturer as well as a previous report of Huntington disease plasma biomarker discovery (Gaughwin et al., 2011), as a reference gene, using the comparative Delta Ct method. The ΔCt value is the difference between the Ct of the target microRNA and that of the reference microRNA (ΔCt = Ct miR34 × − Ct miR16). To include the spike-in results in this calculation, the Ct values were first normalized against *Cel-39* and then against miR-16, according to the equation: normalizing factor = median (Avg.Ct^*Cel*all^ − Avg.Ct^*Cel*sample^); normalized Ct^sample^ = Ct^sample^ + normalizing factor (Livak and Schmittgen, 2001). Correlation between MMSE scores and expression levels of miR-34c and miR-34a in AD and NEC patients was calculated using the Pearson correlation coefficient (r) (Taylor, 1990); a correlation coefficient value of 0.8 or above indicates strong correlation, whereas a value around 0.5 is indicative of moderate correlation.

Receiver-Operating Characteristic (ROC) curves were used to determine the accuracy of the test differentiating AD from NEC individuals for miR-34c and miR-34a in age-matched cohorts (Zweig and Campbell, 1993; Fawcett, 2006). We calculated the sensitivity, specificity and accuracy as follows;

Sensitivity = TP/(TP + FN) = (Number of true positive assessments)/(Number of all positive assessments);
Specificity = TN/(TN + FP) = (Number of true negative assessments)/(Number of all negative assessments)
Accuracy = (TN + TP)/(TN + TP + FN + FP) = (Number of correct assessments)/(Number of all assessments)

A true positive (TP) is defined as an individual showing concordance between disease presence and a diagnostic test result, while a true negative (TN) represents disease absence with test result also negative. Contrary to these scenarios is discordance between these two categories: i.e., a false positive (FP) is defined as disease absence in an individual whose diagnostic test is positive, and a false negative (FN) characterizes disease presence in an individual whose diagnostic test is negative. All four counts, i.e., TP, TN, FP, and FN, were calculated with a cut-off point base (Li and Chung, 2013).

## Results

### Selection of samples for best RNA quality, and controls for calculation of miR-34a and miR-34c levels

**Figure 1:**
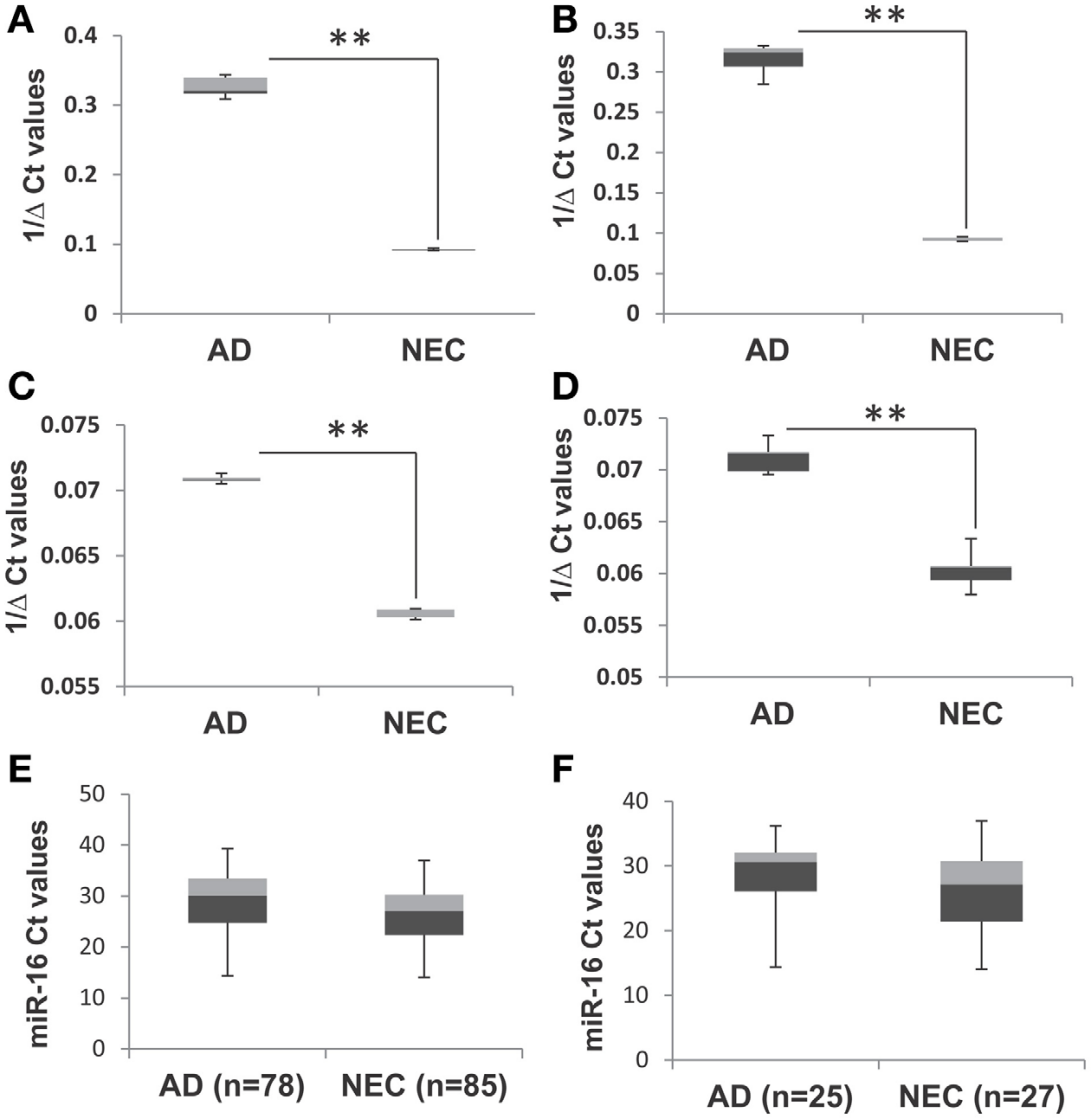
**Box plot representation of data from spike-in experiment with AD and NEC samples, miR-34c, with spike-in (A) and without spike-in (B) of *Cel-39*; and miR-34a with spike-in (C) and without spike-in (D)**. These figures show that levels of miR-34a and miR-34c are similar with and without spike-in experiments in both Alzheimer disease (AD) and normal elderly control (NEC) plasma samples. The Ct values of miR-16 in AD and NEC samples are represented as box plots in the whole population **(E)** and age-matched cohorts **(F)**. These figures indicate that levels of miR-34a and miR-34c are of the same range in both total and age-matched cohorts among both AD and NEC plasma samples. Student's *t-*test was used to determine statistical significance; ^**^P < 0.01.

**Figure 2:**
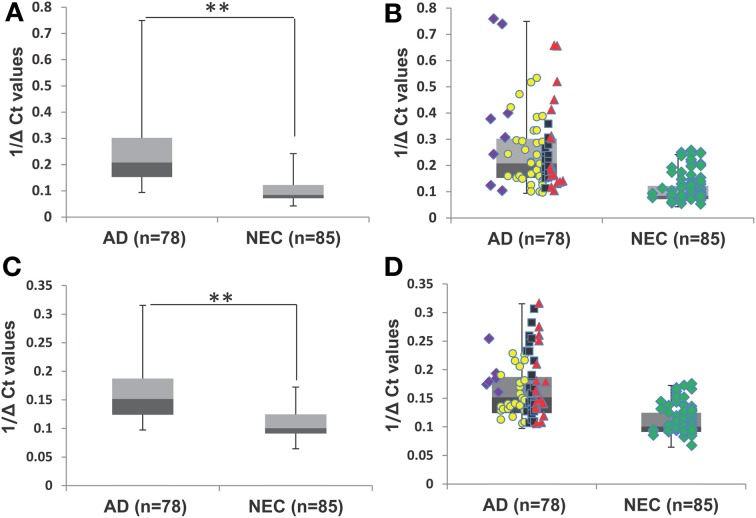
**Transcript levels of miR-34a and miR-34c in plasma samples of Alzheimer's disease (AD) patients and normal elderly controls (NEC). (A)** Comparison of plasma levels of miR-34c in the whole population of AD patients (*n* = 78) with the NEC cohort (*n* = 85). **(B)** Distributions of individuals' levels of miR-34c along the box plot. NEC individuals were distributed in a close cluster around the median levels of the box plots, whereas AD specimens were distributed over a wider range with higher standard deviation. **(C)** Box plot representation of comparison in plasma levels of miR-34a in the whole population of AD patients (*n* = 78) with the NEC cohort (*n* = 85). **(D)** Individual miR-34a values along the box plot are less wide spread in the AD group as compared to those for miR-34c and a tight cluster for NEC is also observed. The data are plotted as inverse ΔCt derived from qPCR analysis. Data points of Mini-Mental Status Examination (MMSE) score ranges are: 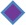 severe AD (score 4–9), 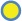 moderate AD (score 10–20), 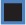 mild AD (score 21–24), 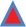 AD outlier (score 25–29), 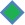 NEC (score 25–30). Student's *t-*test was used to determine statistical significance; ^**^P < 0.01.

From blood samples of 110 AD patients and 123 NEC, only 78 AD and 85 plasma specimens were selected to meet our requirements for RNA integrity: a single peak of small RNA in the 4–40 nucleotide (nt) range (Figure 1S). The age range for the AD group is between 56 and 90, with an average age of 80; the NEC cohort is between 61 and 90, with an average of 72 (Table 1). Our cohort's cognitive ability, assessed by the MMSE, is as reported in the literature (Sharp and Gatz, 2011), i.e., lower scores are linked with fewer years of education in AD individuals (Figure 2S). Our recruitment program for normal control individuals is limited by the fact that many are in their 60 s. In order to achieve an age-matched study design, we further selected from the AD and NEC groups such that the age range of both subcohorts lies between 76–90; the average age for the former is 82, and the latter 80 (Table 1).

**Table 1:** **Plasma specimens from the entire cohorts of Alzheimer's disease (AD) and normal elderly control (NEC) individuals, selected after RNA quality control, with age range, average age with standard deviation (SD), and sample size (N) for acceptable RNA in the entire cohort and age-matched cohorts**.

	**Original**	**Acceptable RNA**			**Age-matched**		
	***N***	**Age range**	**Avg (*SD*)**	***N***	**Age range**	**Avg (*SD*)**	***N***
AD	110	56–90	80 (6.17)	78	76–90	82 (4.11)	25
NEC	123	61–90	72 (1.31)	85	76–90	80 (3.26)	27

To optimize the accuracy of quantitative PCR assays, we used two control steps: (a) quality control for the cDNA synthesis step with *C. elegans* Cel 39 spike-in; and (b) using miR-16 to standardize baseline level determination. Figures 1A–D shows similar levels of miR-34a and miR-34c with and without spike-in experiments in both AD and NEC plasma samples. These results suggest that the protocol established for miR-34a and miR-34c qPCR assays is stringent, providing optimal quality cDNA synthesis with minimal putative RT-RNA inhibitions. An additional standardized control is selecting appropriate microRNAs with no changes between AD and control group blood samples. We followed the manufacturer's recommendation, as well as reports by Kroh et al. (2010) and Gaughwin et al. (2011) to use miR-16 as the control for each individual qPCR assay. However, since miR-16 is an abundant microRNA associated with red blood cells, even minimal hemolysis can cause unreliable qPCR results, as shown by Müller et al. (2014) in cerebrospinal fluid (CSF) qPCR assays. In this context, we evaluated miR-16 levels in all samples used in our study, as shown in Figures 1E,F, and found that in both total and age-matched cohorts, levels of this microRNA are similar between the AD and NEC cohorts. Thus, miR-16 levels are not differentially regulated between AD and NEC plasma samples in our study, and can serve as a standard.

### Expression levels of miR-34a and miR-34c in plasma samples of AD patients and normal elderly controls (NEC)

Our initial study of quantitative reverse transcription PCR (qPCR) analyses of transcript levels of miR-34a and -34c in plasma samples of AD and NEC was performed with the entire cohorts (Table 1). When miR-34c levels were analyzed, NEC individuals were distributed in a close cluster around the median levels of the box plots, while AD specimens were distributed over a wider scatter span, with significantly wider SD range (Figures 2A,B). Similar wide spreads of AD values while NEC samples are clustered were also observed with miR-34a levels (Figures 2C,D). Table 2 shows the wide distribution and high SD indices for both microRNAs in the AD group. To address the concern that higher AD microRNA values could be due to the older age group used, we next selected from our cohorts NEC individuals age-matched to AD counterparts, as listed in Table 1.

**Table 2:** **Plasma microRNA 1/deltaCt level ranges in Alzheimer disease (AD) patients and normal elderly controls (NEC) with standard deviation *(SD)* and median, for miR-34c and miR-34a in the whole cohorts**.

**Whole cohort**	**miR-34c**		**miR-34a**	
	**Range (*SD*)**	**Median**	**Range (*SD*)**	**Median**
AD	0.09–0.75 (0.15)	0.21	0.09–0.32 (0.05)	0.15
NEC	0.04–0.24 (0.05)	0.08	0.06–0.17 (0.02)	0.1

**Figure 3:**
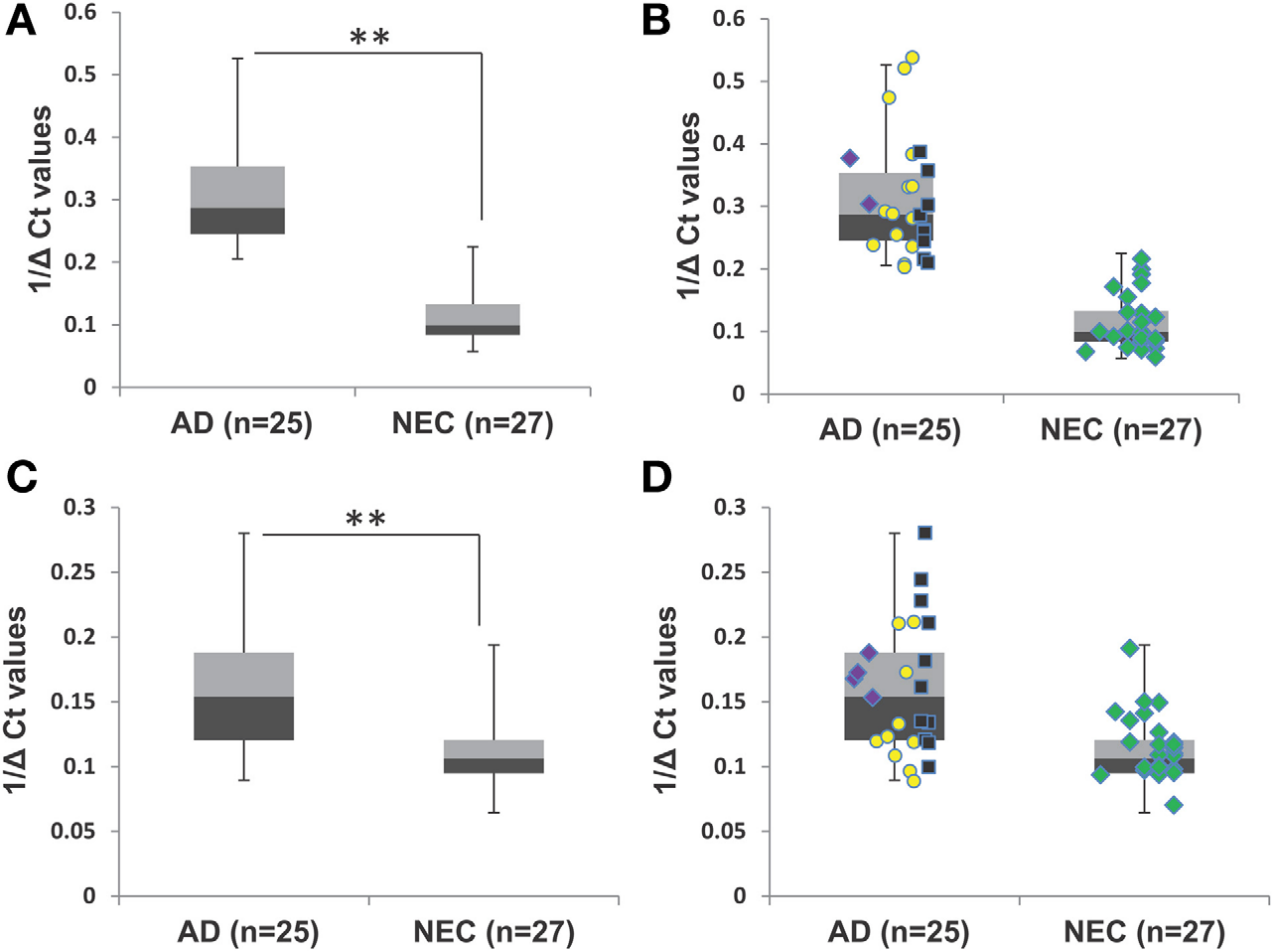
**Transcript levels of miR-34a and miR-34c in plasma samples of age-matched cohorts of Alzheimer's disease (AD) patients and normal elderly controls (NEC). (A)** Plasma level comparison of miR-34c in the age-matched cohort of AD patients (*n* = 25) with the NEC cohort (*n* = 27). AD plasma samples in the age-matched cohort showed higher miR-34c levels than controls. **(B)** Distribution of individuals' levels of miR-34c along the box plot; NEC individuals were distributed in a close cluster around the median of the box plots, while AD patients were distributed over a wider range with greater standard deviation. **(C)** Comparison of plasma levels of miR-34a in age-matched cohorts of AD patients (*n* = 25) with the NEC cohort (*n* = 27). miR-34a levels were distributed in a range that is not differentially expressed between AD and NEC groups, with more spread-out distribution for both groups, as well as significant overlapping ranges, by both box and scatter plots. Distributing individuals along the box plot, NEC were distributed in a close cluster around the median levels of the box plots, whereas AD specimens were distributed over a wider range with higher standard deviation. Data points represent Mini-Mental Status Examination (MMSE) score as follows, (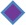) severe AD (4–9), (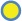) moderate AD (10–20), (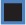) mild AD (21–24), and (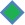) NEC (25–30). **(D)** Representation of individual miR-34a levels along the box plot for the range of distribution between AD and NEC groups. The data are plotted as inverse ΔCt derived from qPCR analysis. Student's *t-*test was used to determine statistical significance; ^**^P < 0.01.

Figures 3A,B shows box plots and distribution of individual values for miR-34c in the two age-matched subcohorts. Clearly, the majority of AD plasma specimens in the age-matched control study exhibit higher miR-34c levels than do normal controls; Table 3 shows 1/deltaCt values in the 0.20–0.53 range for AD, and 0.05–0.22 for NEC plasma specimens. When levels of miR-34a were analyzed, however, the range of distribution is not differentially expressed between the AD and NEC groups, with more spread-out distribution for both groups, as well as significant overlapping ranges, by both box plot and scatter plots (Figures 3C,D; Table 3). Levels of miR-34b were evaluated in the same age-matched subcohorts of the AD and NEC groups in plasma; the results show no significant difference between the two groups (Figure 3S, Table 1S). The box plot presentation of inverted ΔCt values from AD and NEC groups shows no change, indicating that the level of miR-34b does not change in the AD group compared to NEC.

**Table 3:** **Ranges of 1/deltaCt levels of plasma microRNAs in Alzheimer disease (AD) patients and normal elderly controls (NEC), with standard deviation *(SD)* and median, for miR-34c and miR-34a in age-matched subcohorts**.

**Age-matched cohort**	**miR-34c**		**miR-34a**	
	**Range (*SD*)**	**Median**	**Range (*SD*)**	**Median**
AD	0.20–0.53 (0.09)	0.29	0.08–0.28 (0.05)	0.15
NEC	0.05–0.22 (0.05)	0.1	0.06–0.19 (0.03)	0.11

**Figure 4:**
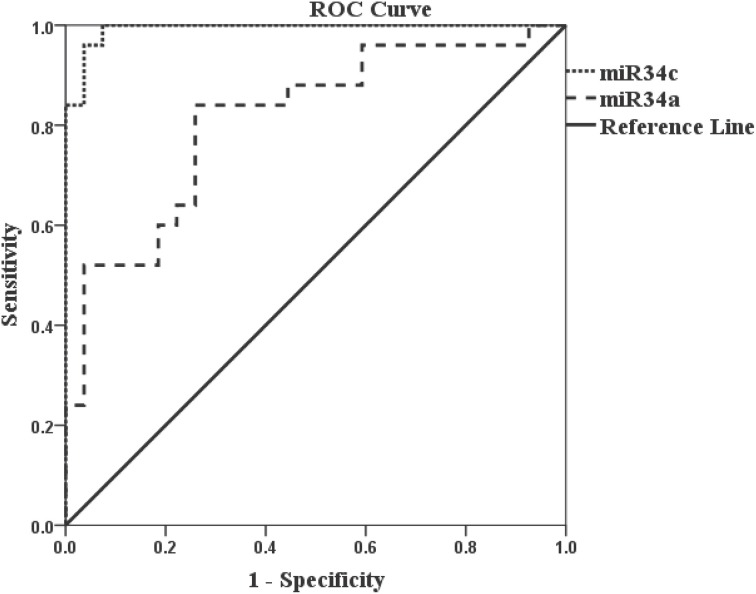
**Receiver Operation Characteristic (ROC) curves representing AD diagnostic tests by miR-34c and miR-34a in age-matched cohorts**. The Receiver Operation Characteristic (ROC) curve for miR-34c (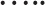) is an excellent test since the area under the curve is 0.99 (*p* < 0.0001), number of samples for AD = 25 and NEC = 27. The area under the Receiver Operation Characteristic (ROC) for miR-34a (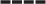) is 0.81 (*p* = 0.0001) which is a good/fair test, number of samples for AD = 25 and NEC = 27. Sensitivity and specificity are reported based on a cut-off point chosen using the coordinates of Receiver Operation Characteristic (ROC) curves with balanced levels of high sensitivity and specificity. The diagonal line represents a reference line showing zero sensitivity and zero specificity (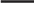).

Accuracy in differentiating AD (*n* = 25) and NEC (*n* = 27) individuals by miR-34c levels in plasma specimens was further tested by ROC curves (Figure 4). The area under the miR-34c curve is 0.99 (*p* < 0.0001), essentially 1.0, indicating perfect accuracy of the data; 95% confidence interval values fall in the range of 0.97–1.0. The ROC curve shows that miR-34c level is an excellent test for AD, with 94% accuracy, 92% sensitivity, and 96% specificity (Table 4). The area under the ROC curve for miR-34a is 0.81 (*p* = 0.0001), indicating a fairly good test; the 95% confidence interval lies between 0.69 and 0.93. The sensitivity is 84%, and specificity is 74% (Figure 4, Table 4). The area under the ROC curve, sensitivity, specificity and accuracy were calculated for a cut-off point chosen using the coordinates of ROC curves with balanced levels of high sensitivity and specificity.

**Table 4:** **Receiver Operating Characteristic (ROC) curve parameters for plasma miR-34c and -34a among age-matched subcohorts**.

**ROC curve**	**miR-34c**	**miR-34a**
Area under curve (AUC)	0.99 (*p* < 0.0001)	0.81 (*p* = 0.0001)
Sensitivity	0.92	0.84
Specificity	0.96	0.74
Accuracy	0.94	0.79

### miR-34c levels in moderate and mild AD compared with age-matched normal elderly controls

**Figure 5:**
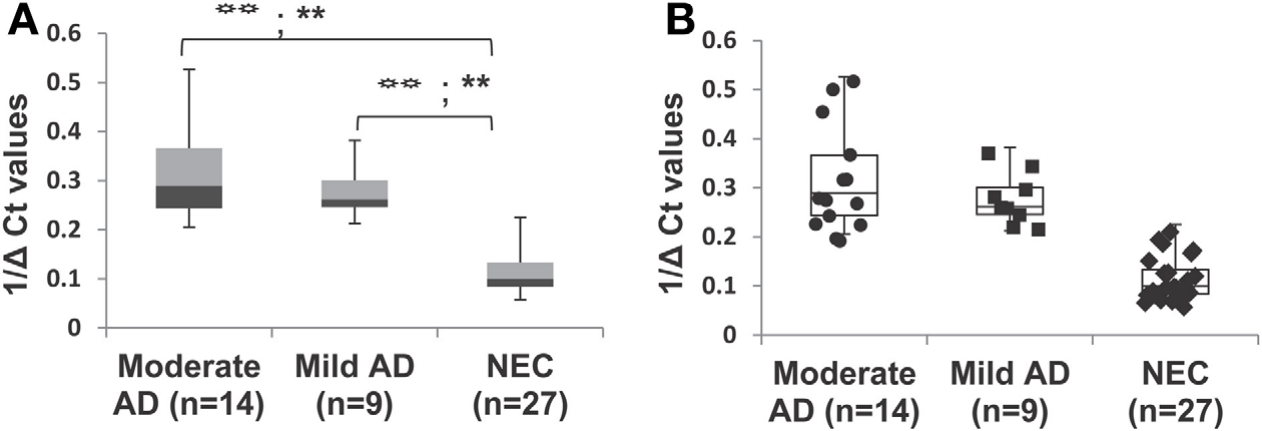
**Differential expression of miR-34c in plasma from patients with mild and moderate severity of Alzheimer's disease (AD), compared with normal elderly controls (NEC) in age-matched cohorts**. AD patients were grouped by mild and moderate severity according to their Mini-Mental Status Examination (MMSE) scores. **(A)** Percentile distribution of samples within the various stages of AD and NEC, plotted against inverse ΔCt for individuals of age-matched cohorts. The levels of miR-34c are higher in samples from moderate (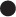) and mild (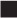) clinical groups, with MMSE scores in the range of 10–20 or 21–24, respectively, than NEC (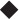) controls in the range of 25–30. **(B)** Data points added to illustrate the distribution of individuals within each group of the age-matched cohorts. We observe NEC to be in a close cluster around the median, whereas AD patients are scattered in a wider range in both moderate and mild groups. Both the LSD test (higher type 1 Error) and the Scheffé test (higher stringency) were used to determine statistical significance. The statistical significance level for the LSD test is represented with the symbol (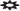), and for Scheffé test is represented with the symbol (∗). 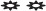
*P* < 0.01, ^**^P < 0.01.

In our age-matched AD cohort, only two patients were evaluated by the MMSE in the severe stage, with scores in the range 4–9. Figures 5A,B show that when these two individuals' samples are excluded from our analysis, miR-34c levels in samples from moderate and mild stages, with MMSE scores in 10–20 or 21–24 ranges respectively, remain higher than NEC controls. Table 5 shows that levels of miR-34c expression are not only distinct between the moderate and NEC groups, but also between the mild group and normal controls, with the former in the range of 0.21–0.38, while the latter shows 0.05–0.22 1/deltaCt values. Similar separation between mild AD patients and the normal control group is not observed for levels of miR-34a in this age-matched cohort study, due to overlapping ranges between these two groups (Table 5).

**Table 5:** **1/deltaCt levels of plasma miR-34c and -34a by clinical category of mild and moderate Alzheimer's disease (AD) and in age-matched normal elderly controls (NEC): range with standard deviation (*SD*) and median**.

	**miR-34c**		**miR-34a**	
	**Range (*SD*)**	**Median**	**Range (*SD*)**	**Median**
NEC	0.05–0.22 (0.05)	0.1	0.06–0.19 (0.02)	0.1
Mild AD	0.21–0.38 (0.06)	0.26	0.10–0.28 (0.06)	0.16
Moderate AD	0.20–0.53 (0.11)	0.29	0.09–0.21 (0.04)	0.12

**Figure 6:**
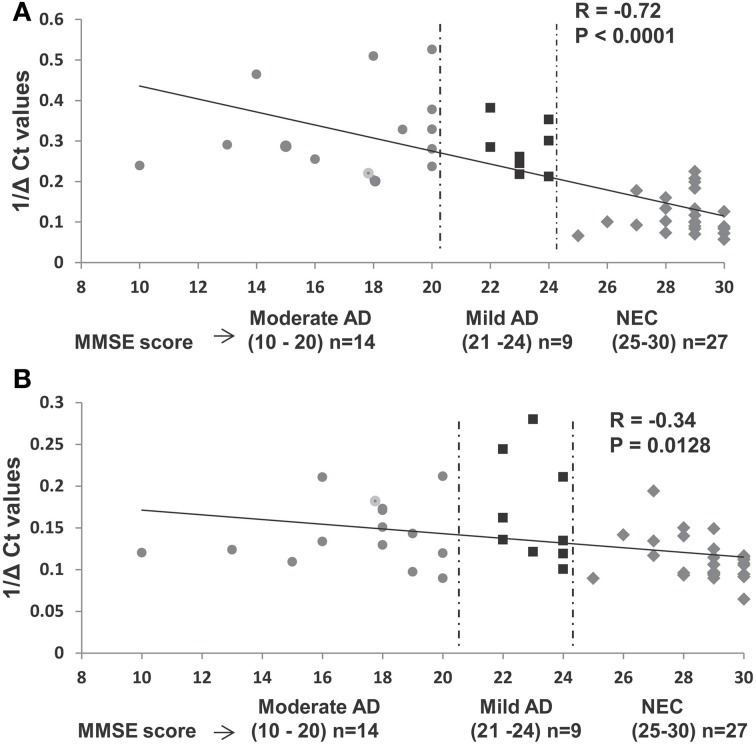
**Relationship between expression levels of miR-34c and miR-34a in plasma with Mini-Mental Status Examination (MMSE) scores in selected samples of age-matched groups. (A)** Plasma expression levels of miR-34c represented by inverse ΔCt, plotted against corresponding MMSE scores. A high Pearson correlation coefficient value of −0.72 indicates a strong correlation between MMSE scores and 1/deltaCt values, *P* < 0.0001. **(B)** Expression levels of miR-34a in plasma of AD and NEC individuals represented by inverse ΔCt, plotted against corresponding MMSE scores. A low Pearson correlation coefficient value of −0.34 indicates a weak correlation between the MMSE scores and 1/deltaCt values, *P* = 0.012. Data points of Mini-Mental Status Examination (MMSE) score are shown as follows: (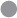) moderate AD (score 10–20), (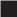) mild AD (score 21–24), and (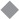) NEC (score 25–30).

We investigated further the relationship between MMSE and miR-34c levels, by calculating Pearson's correlation *r*-value of miR-34c levels and MMSE scores for moderate and mild AD and age-matched control cohorts. Figure 6A shows an *r*-value of −0.72, suggesting a strong inverse correlation between the expression levels of miR-34c and the two cognitive assessment groups. This inverse correlation between MMSE scores and miR-34c levels, i.e., lower scores and higher levels, as shown for the three groups, was not observed for miR-34a expression, with an r coefficient score of −0.34 (Figure 6B). Likewise, when Pearson's correlation was computed for only the moderate and mild clinical groups without NEC, the *r*-value is very low. These results suggests no relationship between MMSE scores and the levels of the two microRNA expression when comparing the moderate and mild groups between themselves (Figure 4S).

### Increased expression of miR-34c in cellular components of AD blood samples

**Figure 7:**
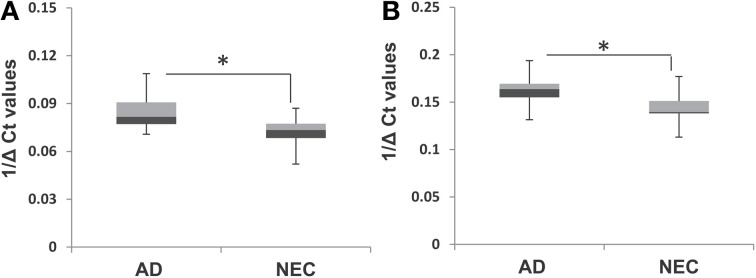
**Box plot representation of transcript levels of miR-34a and miR-34c in peripheral blood mononuclear cell (PBMC) samples from Alzheimer's disease (AD) patients and normal elderly controls (NEC)**. Comparison of levels of miR-34c **(A)** and miR-34a **(B)** in AD patients with that of the NEC cohort in PBMCs, showing increased transcript levels for both microRNAs in the former group, compared to normal controls. Student's *t-*test was used to determine statistical significance; ^*^P < 0.05.

The observation of increased miR-34c expression in AD plasma prompted us to determine the level of expression of this microRNA in the cellular compartment, i.e., PBMCs of age-matched cohorts of AD and NEC samples. Increased miR-34c levels were observed in PBMC samples of AD blood specimens, compared to normal controls (Figure 7A), with a similar increase for miR-34a in AD over normal controls (Figure 7B). However, as shown in Table 6, as observed in the plasma samples, there is scant range overlap for miR-34c levels between AD and age-matched controls; this is not true for miR-34a levels between the two groups. Interestingly, the miR-34a levels assayed here as validation for our previous study (Schipper et al., 2007) show the same range of fold changes, i.e., ~3-fold increase in AD samples beyond normal controls (Figure 5S).

**Table 6:** **1/deltaCt level ranges, with standard deviation (SD) and median, of microRNAs in PBMC from Alzheimer's disease (AD) patients and age-matched normal elderly controls (NEC), for miR-34c and miR-34a**.

**PBMC**	**miR-34c**		**miR-34a**	
	**Range (*SD*)**	**Median**	**Range (*SD*)**	**Median**
AD	0.07–0.11 (0.01)	0.08	0.13–0.19 (0.01)	0.16
NEC	0.05–0.08 (0.01)	0.07	0.11–0.17 (0.02)	0.14

### Functional analysis of the repression of target proteins by overexpressing miRs -34a and -34c in cultured cells

**Figure 8:**
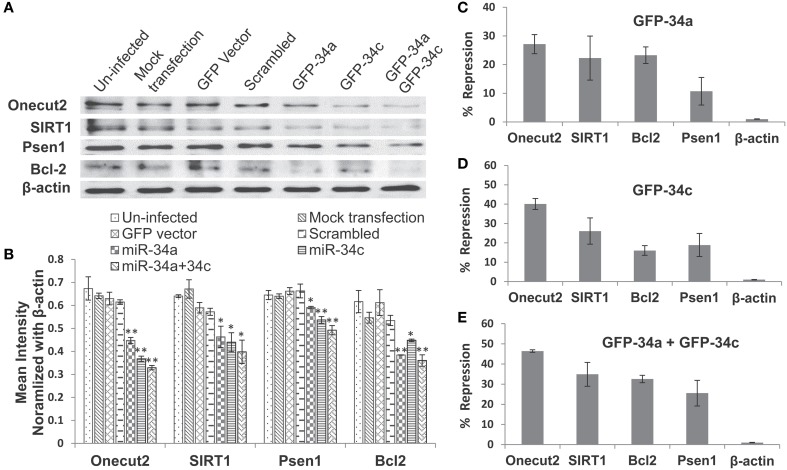
**Repression of expression of selected candidate target genes of miR-34a and miR-34c by transfection analysis**. Human embryonic kidney (HEK 293) cultures were transfected with green fluorescence protein (GFP) expression vectors containing sequences encoding miR-34a, miR-34c, or a scrambled sequence. Controls included un-infected cultures, mock transfected cultures, cells transfected with either GFP vector alone or carrying a computer-generated scrambled sequence. **(A)** Western blot analysis of cell lysates probed for SIRT1, Onecut2, Presenilin-1, Bcl2, and β-actin proteins; **(B)** Graphic representation of a comparison of band intensities between the various treatments; image intensities of Western blotted bands were normalized against the β-actin band, which is constant throughout. Graphic representation showing percent of repression, estimated using scrambled controls for comparison (panels **C–E**): transfection with miR-34a **(C)**; miR-34c **(D)**; miR-34a and miR-34c **(E)**. One-way ANOVA followed by LSD test was used to determine statistical significance; ^*^P < 0.05, ^**^P < 0.01.

We next investigated whether miR-34c represses the same targets as reported for its sister, miR-34a (He et al., 2007; Yamakuchi et al., 2008), by transfection studies in cultures of human embryonic kidney cells (HEK 293) overexpressing these microRNAs. Only cultures with >98% of cells showing green fluorescence positivity for the green fluorescence protein gene (GFP) were used in our functional assays. Our results with these transfection experiments show repression of Psen1, Bcl2, Sirt1, and Onecut2 by miR-34a ranging from ~11% to 27%, with the latter being the most affected (Figures 8A–C, Table 7). A more pronounced impact is observed by miR-34c, with repression ranging from ~16 to 40% (Figures 8A,B,D, Table 7). Co-transfection with both miRNAs induced further repression of all four target proteins, from ~32 to 46% (Figures 8A,B,E, Table 7). In all analyses, repression levels were evaluated by comparing transfected and scrambled control cultures, with repression of actin observed at ~0%. These observations suggest that miR-34c is a stronger suppressor of target gene expression than miR-34a, and that Onecut2 is the most repressed among the target proteins.

**Table 7:** **Levels of miR-34a and miR-34c repression of four target genes' expression**.

	**miR-34a *vs*. scrambled**	**miR-34c *vs*. scrambled**	**miR-34a + 34c *vs*. scrambled**
**Protein**	**Mean % repression (*SD*)**	**Mean % repression (*SD*)**	**Mean % repression (*SD*)**
Onecut2	27.15 (3.34)	40.13 (2.85)	46.42 (0.63)
SIRT1	22.28 (7.68)	26.09 (6.78)	34.89 (5.92)
Bcl2	23.27 (2.87)	16.06 (2.48)	32.57 (1.85)
Psen1	10.69 (4.8)	18.89 (5.94)	25.54 (6.39)
β-actin	0	0	0

Percent repression of target proteins in HEK 293 cells transfected with miR-34a, miR-34c, or both miRNAs. The repression levels shown here represent protein band intensities, after normalization against β-actin band intensities, when cultures transfected by microRNA are compared with those transfected by scrambled controls. As shown in Figure 8, the % change of β-actin levels between cultures transfected by the microRNAs of interest and by the scrambled controls is ~0. Mean % repression with standard deviation (SD) of each miR for each protein is tabulated.

## Discussion

Members of the microRNA-34 family are present in most tissues, including PBMC. Our previous study reported miR-34a as a lead microRNA from array profiling and validated by qPCR assays, showing a 2.5-fold increase in PBMC isolated from AD specimens (Schipper et al., 2007). The present study shows that this microRNA's sister, miR-34c, is even more prominently increased in both PBMC and plasma fractions of AD blood samples over age-matched NEC. Most importantly, the majority of miR-34c levels among mild AD patients, determined by mini-mental state examination (MMSE), are elevated beyond those of normal controls. In addition, inclusion of patients with moderate degrees of AD dementia with mild and normal counterparts in our study shows a relatively strong inverse relationship between MMSE scores and levels of this microRNA among the three groups. Levels of miR-34b, the bicistronic sister of miR-34c, are not significantly different between AD and NEC age-matched controls. Thus, our results reported here will serve as future leads to identify key microRNA abundance, such as miR-34c, as noninvasive indicators for plasma changes associated with AD.

Age-matched circulating blood microRNA studies like ours by design suffer several limitations, such as: (1) restriction of obtaining gene expression changes at a particular snap-shot time window; and (2) inability to link with changes of the same gene expression in individuals' brains. The first limitation cannot be addressed by small scale studies like ours, but rather in large consortium studies such as the Alzheimer's Disease Neuroimaging Initiative (ADNI) study of the National Institute on Aging, USA, with longitudinal blood sample collection linking plasma and CSF analysis study, and MRI imaging of brain changes in the same individuals. However, most of these studies are centered on the analysis of protein changes, with some focus on Tau and Aβ as biomarkers (O'Bryant et al., 2011; Toledo et al., 2011, 2013). Other studies such as Müller et al. (2014) have limited sample size because such samples are only available from a very few centers where longitudinal *ante mortem* blood or CSF samples are collected along with follow-up acquisition of autopsy brain specimens. Comparative studies between *ante-mortem* blood samples and *post mortem* autopsy will emerge in future studies, yielding results elucidating the relationships between plasma biomarkers and brain pathology structural changes, including but not limited to amyloid plaques and Tau-associated tangle formation.

To identify body fluid-associated microRNA biomarkers for neurodegeneration, standardization of the sample collection protocol and quality control of RNA isolation are two obvious quality control criteria in any study of this kind. Although we have established meticulous procedures for collecting blood samples and further processing to PBMC and plasma fractions, a significant portion, ~20%, of the RNA fractions isolated from these samples were unacceptable because of poor RNA integrity. Even among the 70–80 samples with good RNA quality, we had to narrow our study to 25 AD and 27 NEC as shown in Table 1, in order to satisfy age-matched criteria for our study. With samples from these two subcohorts, as in most neurodegenerative studies, before proceeding to qPCR assays of a particular microRNA of interest, we implemented quality control to insure the cDNA synthesis part of the assays by incorporating *Cel 39* spike-in in the step of RNA isolation.

Besides the above issues, selection of reference microRNAs for determination of microRNA levels of expression is crucial for studies of blood samples of neuronal disorders. We use miR-16 as our reference microRNA, following the report by Gaughwin et al. (2011), who used it as the reference microRNA to show that miR-34b is elevated in the plasma of Huntingdon mutation gene carriers, prior to disease manifestation. Interestingly, our study with this microRNA shows that miR-34b levels do not differ significantly in plasma specimens between AD patients and NEC. A recent study by Müller et al. (2014) shows that miR-16 and miR-146 are increased in AD in both hippocampus and cerebrospinal fluid (CSF). However, this report also suggests that miR-16, a major red blood cell microRNA (Kirschner et al., 2011, 2013; McDonald et al., 2011), may be contaminatedby hemolysis in CSF samples; even as little as 100 μl of erythrocytes added skews the qPCR results (Müller et al., 2014). In addition, several other microRNAs reported so far as unchanged in AD tissue have been used as references to determine levels of microRNAs of interest (Geekiyanage et al., 2012; Sheinerman et al., 2012; Sheinerman and Umansky, 2013). Notwithstanding all these reports, no unifying approach of selecting appropriate reference controls has yet been reached. At present we are limited to the use of miR-16 as the reference, based on the rationale that we found no significant variance in it among our samples, as shown in Figures 1E,F.

The widespread variance in levels of both miR-34c and its sister, miR-34a in AD plasma compared with the tight NEC cluster range, shown in Figures 1–4, clearly suggests individual variability in the control of these microRNAs' expression. From our small age-matched cohorts, miR-34c levels may be a better candidate than miR-34a and miR-34b for future large-scale study as a blood-based biomarker, simply because its expression in most mild AD patients shows higher levels than most NEC. The ROC analyses performed for both miR-34c and miR-34a 1/deltaCt values in age-matched cohorts proved the former to be an excellent discriminant (AUC = 0.99), whereas the latter was merely fairly good (AUC = 0.81). However, these results suggest the need of further research with larger cohorts, to stringently validate our results of sensitivity using miR-34c as a diagnostic biomarker for plasma samples. Likewise, the inverse correlation, observed only when the moderate and mild groups are compared with their NEC between cognitive scoring and levels of expression of miR-34c in plasma also needs future studies with larger cohorts to define their true relationship. Nevertheless, our findings open the possibility that plasma microRNAs, along with other biomarkers, may be fertile ground for future research linking cognitive decline with changes of gene expression in blood samples.

The increased miR-34c in AD plasma may derive from PBMC, where miR-34c is also increased. As reported, microRNAs present in the circulating plasma may be found either in the encapsulated vesicles known as exosomes, or in lipid- or protein-associated free particulates (Smalheiser, 2007; Etheridge et al., 2011). Reports so far are inconclusive as to what proportion of plasma microRNAs are associated with the former vs. the latter components. Future comparative studies of PBMC, exosomal and free-form miR-34c will reveal the relationship among these three components of AD circulating blood specimens responsible for this microRNA's increased expression.

**Figure 9:**
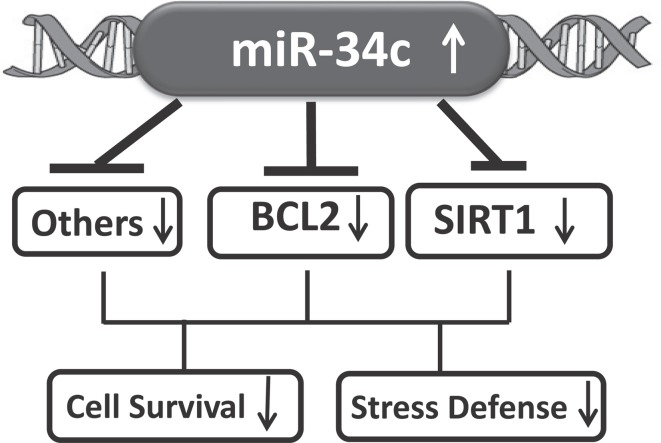
**A model suggesting that increased circulating miR-34c expression represses targets involved in oxidative stress and cell survival signaling pathways**.

MicroRNA-34c, as reported for its sister, miR-34a (He et al., 2007; Yamakuchi et al., 2008) and as shown in Figure 8, represses Bcl2, SIRT1, Psen1, and Onecut2, all associated with cellular survival and oxidative defense signaling (Clotman et al., 2005; Goodall et al., 2013). This led us to suggest that increased miR-34c may be one of many factors contributing to an overall systemic weakening of stress defense and cell survival, as suggested in our model presented in Figure 9. Life-long cumulative oxidative stress induction of p53 up-regulation may activate the expression of this microRNA, with a steadily increasing trend during aging (Li et al., 2011b,c). In AD, this oxidative stress may be further enhanced, manifested in brain as well as perhaps system-wide, reflected in circulating blood by both PBMC and plasma (Maes et al., 2010; Goodall et al., 2013). Future culture experiments in PBMC isolated from AD patients compared with those from normal counterparts will allow us to investigate whether these cells with increased miR-34c levels are indeed prone to apoptotic death, and whether plasma from the same samples stimulates stress and apoptotic signaling in neighboring cells.

In conclusion, data presented here indicate that levels of miR-34c significantly increase in plasma samples of sporadic AD. Future studies with larger age-matched cohorts will validate these results, and reveal whether this microRNA change is characteristic of sporadic AD as a biomarker criterion, and further comparative studies will elucidate whether it is a common biomarker shared among various neurodegenerative disorders, associated with the decline of oxidative defense and cell survival in neuronal dysfunction.

## Disclosure statement

Howard Chertkow is supported by operating grants from the Canadian Institutes for Health Research (CIHR) and the Fonds de la recherche en santé du Québec (FRSQ). Dr. Chertkow sits on an adjudication board for clinical trials for Bristol Myers Squibb, and has been a speaker and Advisory Board member for Pfizer Canada. Hyman M. Schipper is supported by operating grants from the Canadian Institutes for Health Research (CIHR), and has served as consultant to Osta Biotechnologies, Molecular Biometrics, Inc., TEVA Neurosciences, and Caprion Pharmaceuticals. Eugenia Wang is on entrepreneurial leave from the University of Louisville, with 51% of her effort committed to Advanced Genomic Technology, LLC, a start-up company in Louisville, Kentucky; her other 49% is at the University of Louisville, as the Gheens Endowed Chair on Aging and Professor of Biochemistry and Molecular Biology. Shephali Bhatnagar, Zongfei Yuan, Vikranth Shetty, Samantha Jenkins, Timothy Jones, and Eugenia Wang are employees of Advanced Genomic Technology.

### Conflict of interest statement

The authors, except Drs. Howard Chertkow and Hyman Schipper, were all employees of the Advanced Genomic Technology (AGT) in Louisville, Kentucky and the results presented in the paper were generated by the funding support of a Small Business Innovation Technology (SBIR) grant from the National Institutes of Health, USA to AGT.

## Abbreviations

miR or miRNA: microRNA
AD: Alzheimer's disease
NEC: normal elderly control
MMSE: Mini-Mental State Examination
MMSE score (4–9): severe group
MMSE score (10–20): moderate group
MMSE score (21–24): mild group
Bcl2: B-cell lymphoma 2
SIRT1: sirtuin1
Psen1: presenilin-1.
