# Supplementary material for: Increased microRNA-34c abundance in Alzheimer's disease circulating blood plasma
Source: Front Mol Neurosci. 2014 Feb 4;7:2. doi: 10.3389/fnmol.2014.00002 (PMC3912349; doi:10.3389/fnmol.2014.00002)
Supplement: Supplementary file 1 [file DataSheet1.PDF]

Supplemental Table and Figures Including Legends of Bhatnagar, et al.

Table 1S. Ranges of 1/deltaCt values of microRNA (miR)-34b in plasma samples of Alzheimer's disease patients (AD) and normal elderly controls (NEC), with standard deviation (S.D.) and median.

miR-34b in Plasma	AD	NEC
Range (S. D.)	0.18 - 0.35 (± 0.04)	0.20 - 0.30 (± 0.03)
Median	0.26	0.22

Supplemental Figure Legends

Figure 1S. Determining miRNA integrity. The figure represents spectrophotometric scans of purified plasma microRNA, generated by the Agilent 2100 bioanalyzer. Samples 1-4, 6 and 7 were excluded from further analysis, with either high background noise peak or a broad shoulder with noise range beyond the single peak, both indicating low quality RNA. Samples 5 and 8-11 are included in our analysis, and considered good quality RNA with low background. Peaks of various RNA molecular weights are shown in the RNA ladder.

Figure 2S. Demographic distribution of MMSE scores according to education levels in Alzheimer's disease patients (AD) and normal elderly controls (NEC). The figure represents educational level distribution of Alzheimer's disease ● (AD) patients and normal elderly controls ◊(NEC) against their Mini-Mental Status Examination (MMSE) scores.

Figure 3S. Box plot presentation of transcript levels of miR-34b in plasma samples of Alzheimer's disease (AD) patients compared to age-matched normal elderly controls (NEC). No significant difference was observed in the levels of miR-34b between the two groups.

Figure 4S. (A). The expression levels of miR-34c in plasma, represented by inverse ΔCt, plotted against corresponding MMSE scores only among the moderate and mild groups of Alzheimer's disease (AD) patients. A low Pearson correlation coefficient value of -0.09 indicates that there is no correlation between the MMSE scores and 1/ΔCt values. **(B).** Transcript levels of miR-34a in plasma of AD of moderate and mild groups represented by 1/ΔCt, plotted against corresponding MMSE scores only for Alzheimer's disease (AD) patients with a Pearson correlation coefficient value of 0.21. Panel A and B shows that there is no correlation between the MMSE scores and 1/ΔCt values when the moderate and mild groups of AD samples were used for comparison. Data points of Mini-Mental Status Examination (MMSE) score are shown as follows, (●) moderate AD (score 10-20) and (■) mild AD (score 21-24).

Figure 5S. (A) Fold change of about 3.5 in transcript levels of miR-34c; **(B)** ~2.9 fold increase in transcript levels of miR-34a in PBMCs of Alzheimer's disease (AD) patients compared to age-matched normal elderly controls (NEC).

Supplemental Figures

Figure 1S.

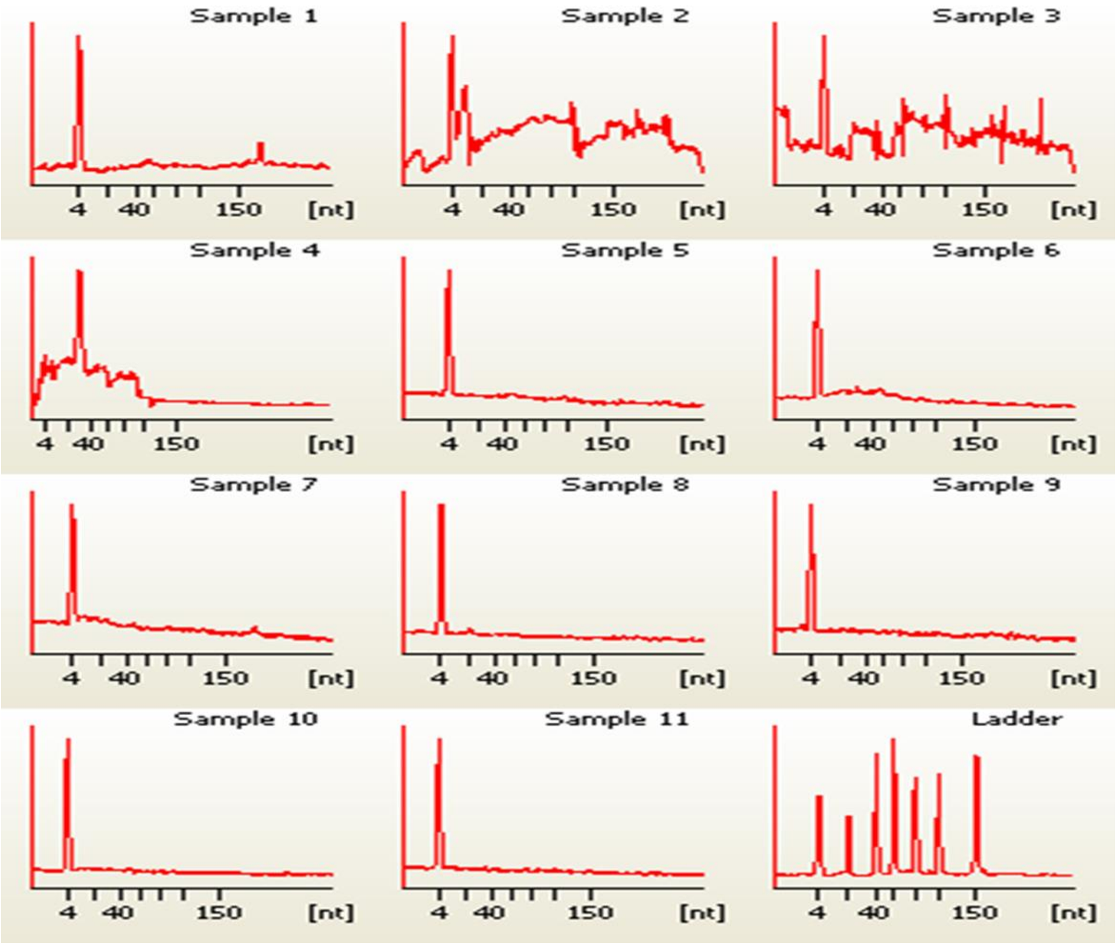

Figure 2S.

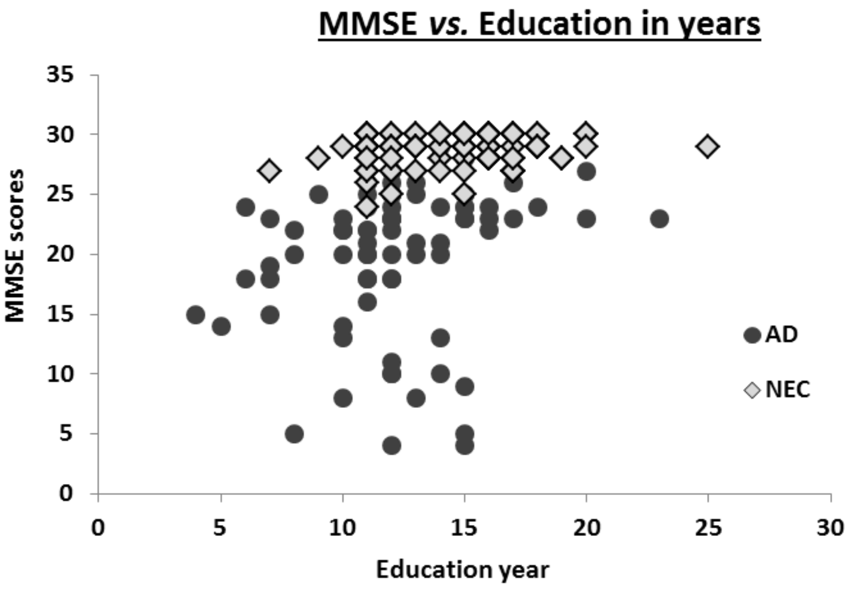

Figure 3S

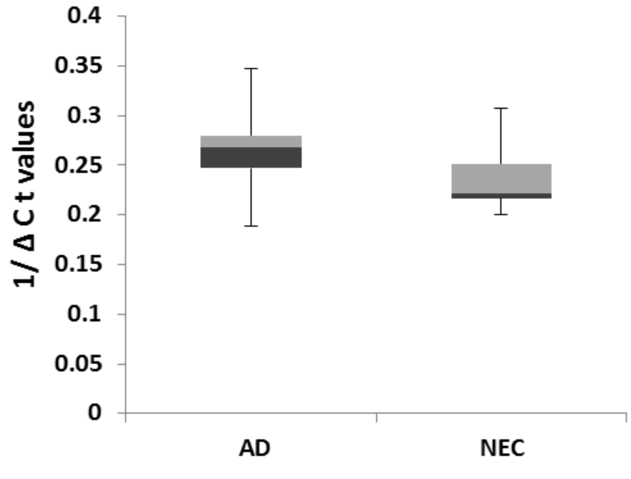

Figure 4S.

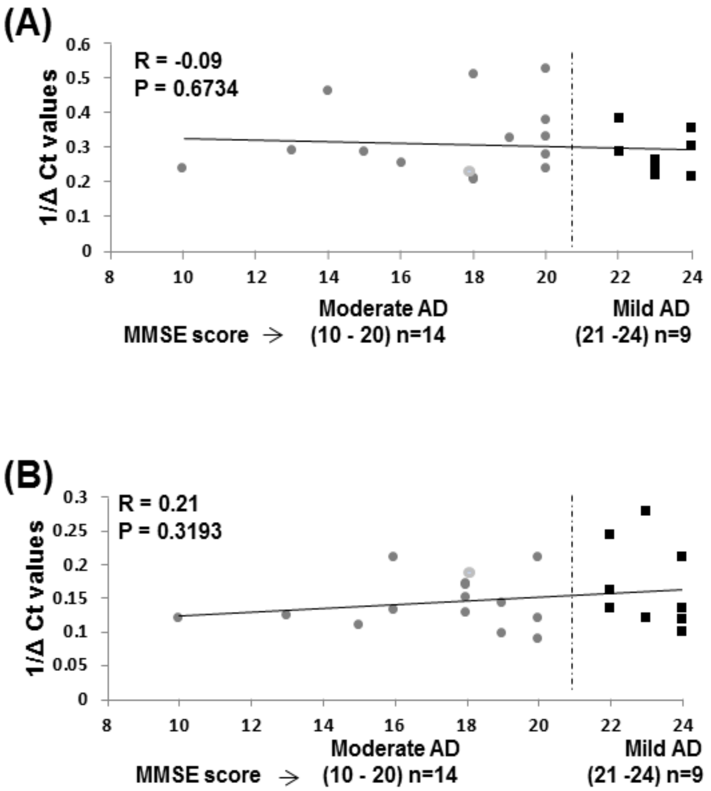
(A)
R = -0.09
P = 0.6734
1/Δ Ct values
Moderate AD
MMSE score →
(10 - 20) n=14
Mild AD
(21 -24) n=9
(B)
R = 0.21
P = 0.3193
1/Δ Ct values
Moderate AD
MMSE score →
(10 - 20) n=14
Mild AD
(21 -24) n=9

Figure 5S.

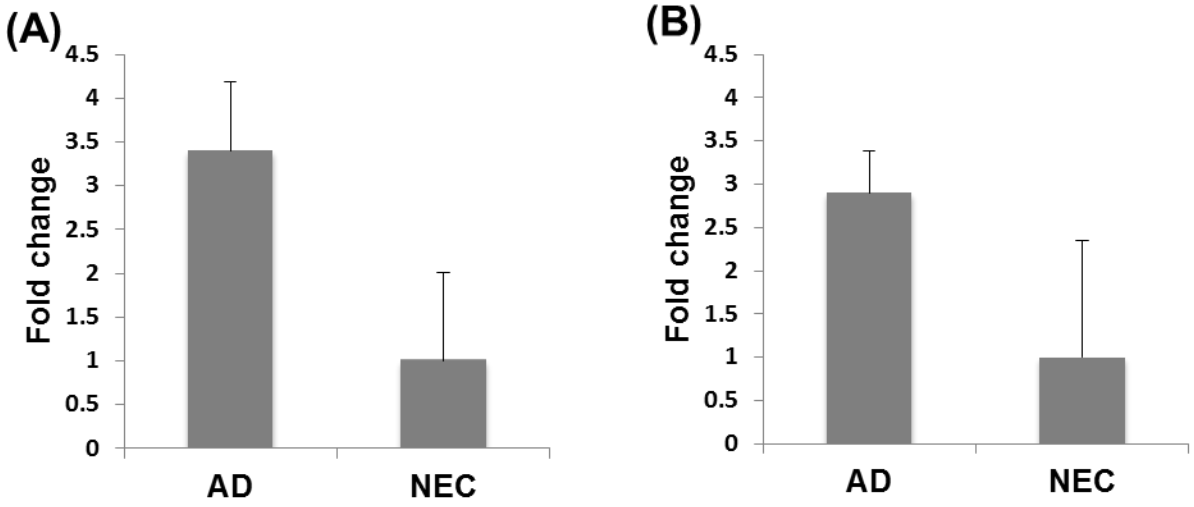
(A)
Fold change
4.5
4
3.5
3
2.5
2
1.5
1
0.5
0
AD
NEC
(B)
Fold change
4.5
4
3.5
3
2.5
2
1.5
1
0.5
0
AD
NEC
